# Supplementary material for: AlleleShift: an R package to predict and visualize population-level changes in allele frequencies in response to climate change
Source: PeerJ. 2021 Jun 15;9:e11534. doi: 10.7717/peerj.11534 (PMC8212829; doi:10.7717/peerj.11534)
Supplement: Supplemental Information 2 [file peerj-09-11534-s002.pdf]

AlleleShift simulation study

Roeland Kindt

22/03/2021

Contents

1 **Packages needed** 1

2 **Obtain simulated data from LEA** 1

3 **Select alleles that are most clearly associated with gradient** 7

4 **Create new simulated data sets** 9
- 4.1 Baseline populations . . . 9
- 4.2 Future populations . . . 12

5 **Calibrate the model** 15

6 **Predict future frequencies** 26
- 6.1 Compare predicted and simulated future frequencies . . . 26
- 6.2 Plot predicted shifts in allele frequencies . . . 31

7 **Discussion** 33

1 Packages needed

```
library(AlleleShift) # also loads BiodiversityR and vegan
library(poppr) # also loads adegenet
library(LEA)
```

2 Obtain simulated data from LEA

Data sets **tutorial.R** and **tutorial.C** in the **LEA** package are subsets of the 4500 neutral SNPs and 500 adaptive SNPs used in the simulation study in Frichot and Francois 2015. The last 50 SNPs of tutorial.C are correlated with the environmental variable available from tutorial.C. These SNPs are selected for further analysis here.

Data are provided for 50 individuals.

The environmental data is first transformed to a range of 0 to 100. Environmental centres along the gradient for baseline populations range between 5 and 75. The populations shift +25 along the range in the future conditions.

```
data(tutorial)
str(tutorial.R)
```

```
##  num [1:50, 1:400] 1 1 2 0 0 2 0 2 1 0 ...
```

```r
str(tutorial.C)
```

```
##  num [1:50] -3.96 -5.12 3.35 -16.99 16.47 ...
```

```r
AlleleData <- tutorial.R[, 351:400]
EnvData <- tutorial.C
summary(EnvData)
```

```
##      Min.   1st Qu.    Median      Mean   3rd Qu.      Max.
## -23.90071 -10.02858   0.50231  -0.09481  11.12218  31.58909
```

```r
summary(EnvData)
```

```
##      Min.   1st Qu.    Median      Mean   3rd Qu.      Max.
## -23.90071 -10.02858   0.50231  -0.09481  11.12218  31.58909
```

```r
EnvData <- 100* (EnvData - min(EnvData)) / (max(EnvData) - min(EnvData))
summary(EnvData)
```

```
##    Min. 1st Qu.  Median    Mean 3rd Qu.    Max.
##    0.00   25.00   43.98   42.90   63.12  100.00
```

Individuals are sorted along the gradient so that the first individual has the lowest value and the last individual has the highest value.

```r
gradient.order <- order(EnvData)
AlleleData <- AlleleData[gradient.order, ]
EnvData <- EnvData[gradient.order]
EnvData
```

```
##  [1]   0.000000   6.413219   8.880776   9.755212  10.724297  10.866225
##  [7]  12.460328  13.400826  14.887664  19.454217  19.821983  19.854725
## [13]  23.974253  28.074904  28.837442  28.901264  30.201894  33.848945
## [19]  35.230482  35.936053  36.401090  36.901425  38.431460  40.789897
## [25]  42.561067  45.393925  45.787849  46.479914  46.695269  47.098588
## [31]  49.103218  50.059571  50.435466  50.888918  54.511087  56.452843
## [37]  62.074801  63.462934  63.963338  64.063853  64.436875  67.775217
## [43]  68.267914  69.736615  72.558699  72.749981  77.435962  78.186955
## [49]  90.840963 100.000000
```

Plots show the counts for the 'A' alleles along the gradient separately for each locus. Whereas the frequency of the A alleles increases clearly along the gradient, there is considerable overlap between occurrences of both alleles over most of the range.

```r
par(mfrow=c(2, 5))

for (i in 1:50) {

plot(AlleleData[, i] ~ EnvData, ann=FALSE)

}
```

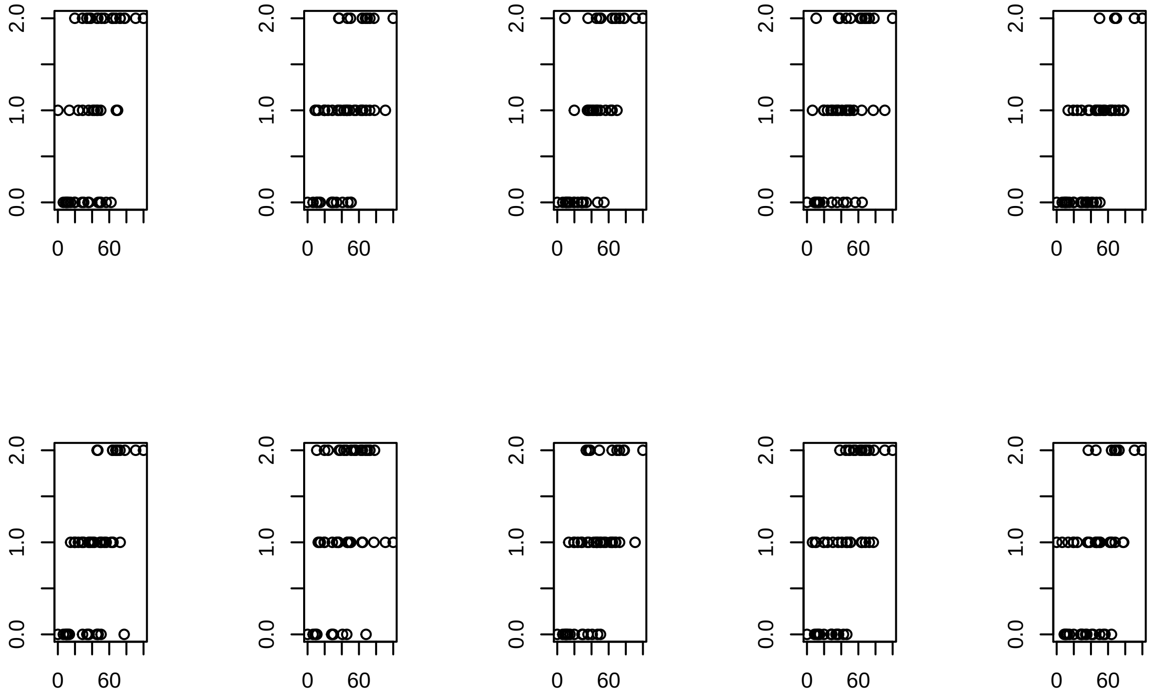
2.0
1.0
0.0
0
60

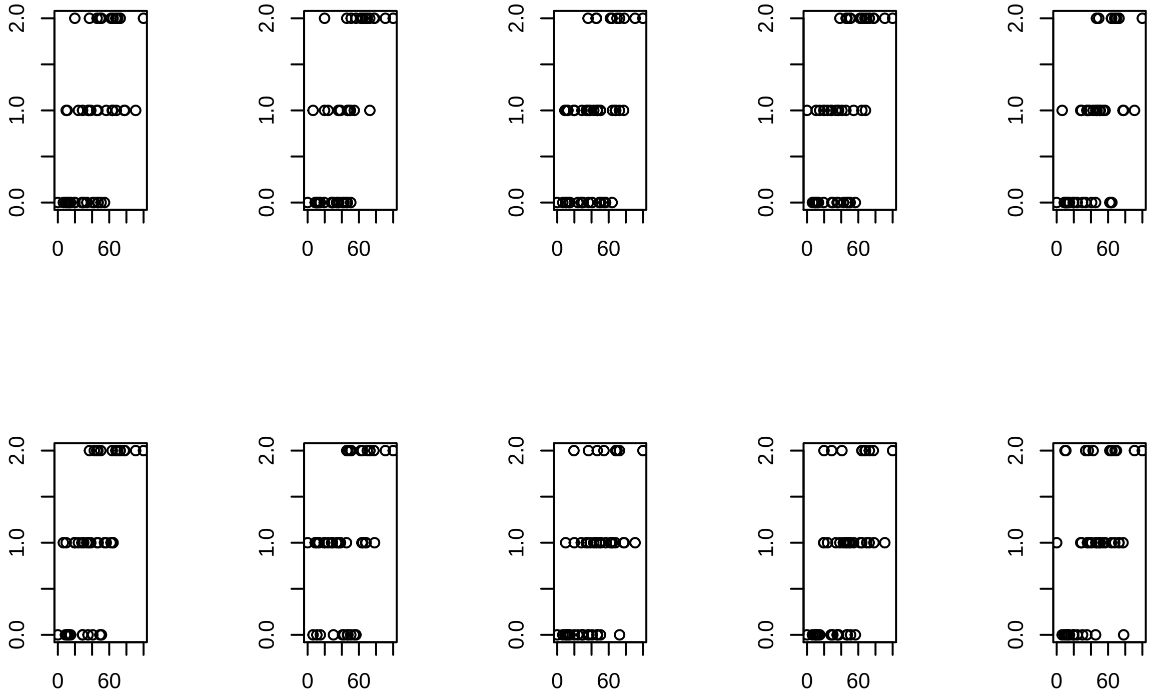
0.0
1.0
2.0
0
60

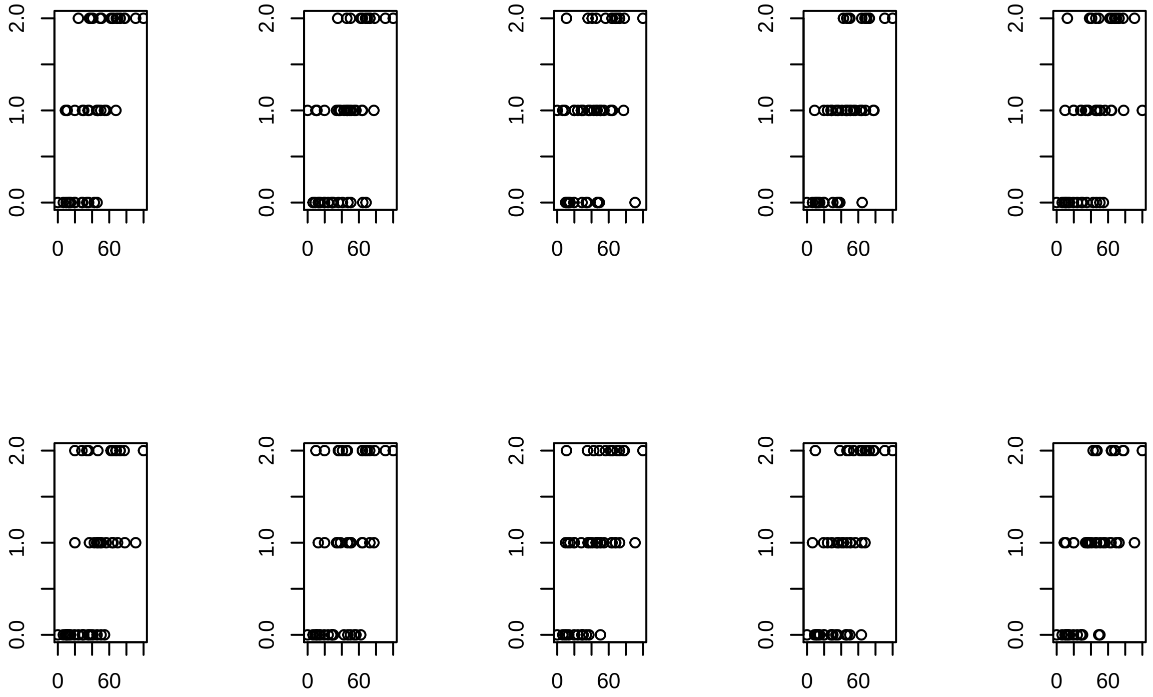
2.0
1.0
0.0
0
60

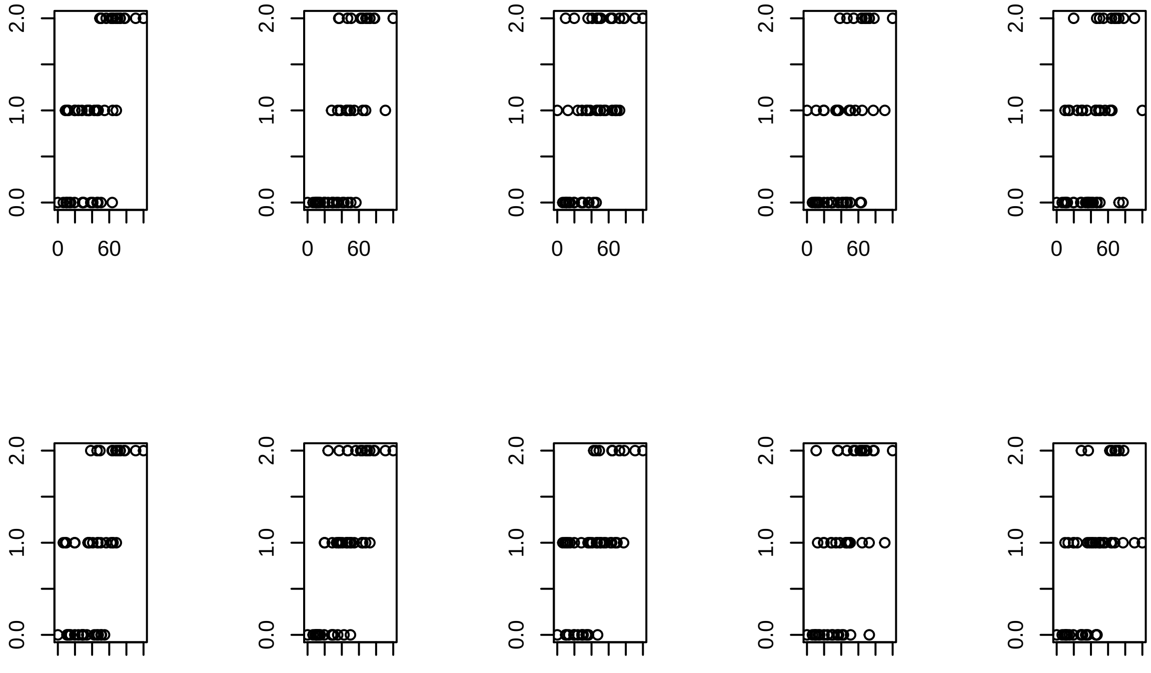

2.0
1.0
0.0
0
60

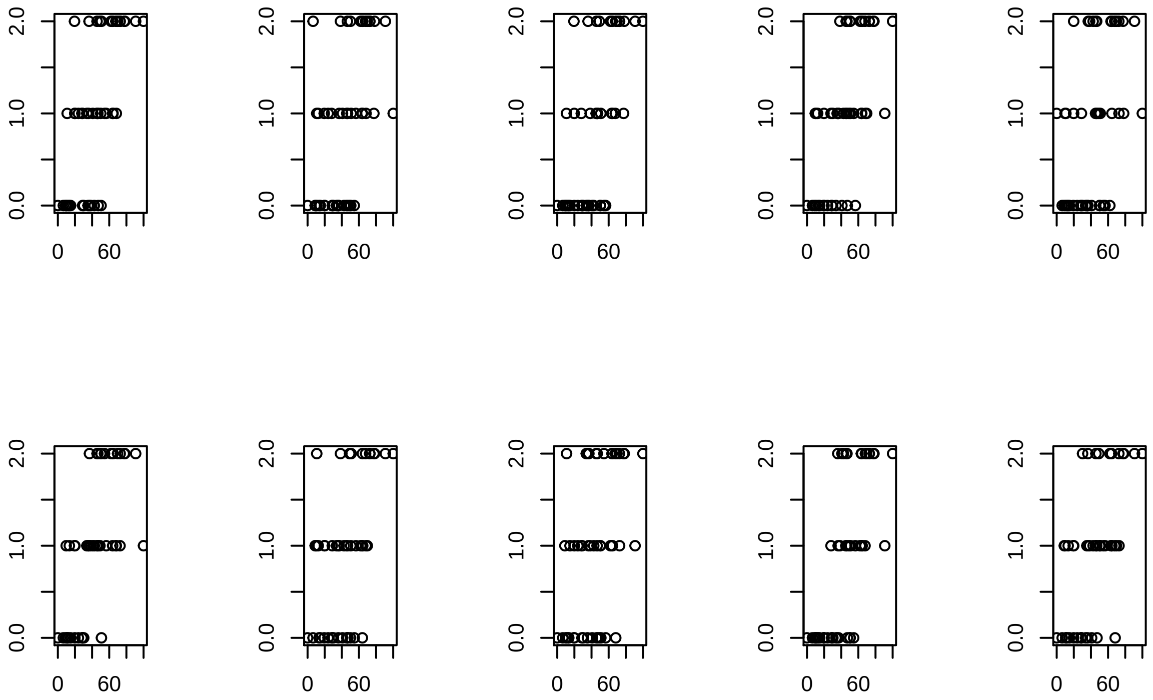


```r
par(mfrow=c(1, 1))
```

3 Select alleles that are most clearly associated with gradient

Via a generalized linear model, the five loci with highest percentages of explained variance are selected.

```r
locus.explained <- numeric(length=50)

for (i in 1:50) {
# i <- 1

  data.i <- data.frame(cbind(A=AlleleData[, i], EnvData))
  data.i$B <- 2 - data.i$A
  data.mod.i <- glm(cbind(A, B) ~ EnvData,
                   data=data.i,
                   family=binomial(link=logit))
  locus.explained[i] <- data.mod.i$deviance / data.mod.i$null.deviance
}

locus.explained[order(locus.explained, decreasing=TRUE)]
```

```
##  [1] 0.8898501 0.8476764 0.8445766 0.8349432 0.8218747 0.8192781 0.8137260
##  [8] 0.8118696 0.8105033 0.7783817 0.7757875 0.7754227 0.7711550 0.7628589
## [15] 0.7602469 0.7556557 0.7436847 0.7432226 0.7396958 0.7355309 0.7335119
## [22] 0.7316037 0.7282626 0.7275067 0.7234654 0.7209665 0.7059418 0.7022914
## [29] 0.6775506 0.6751224 0.6710200 0.6705066 0.6692063 0.6625471 0.6622549
```

```
## [36] 0.6617779 0.6606001 0.6590038 0.6548352 0.6302401 0.6262960 0.6237194
## [43] 0.6203140 0.5799551 0.5762885 0.5670727 0.5640100 0.5616780 0.5353558
## [50] 0.5134911
```

A subset is created with the 5 loci with the clearest trends in frequency changes along the gradient.

```r
AlleleData <- AlleleData[, order(locus.explained, decreasing=TRUE)[1:5]]
```

Plots of the ranges shows that considerable overlap remains among the two alleles. For several loci, there are cases where at the lowest section of the range, both alleles are observed, not only the B allele.

```r
par(mfrow=c(1, 3))

for (i in 1:5) {

plot(AlleleData[, i] ~ EnvData, ann=FALSE)

}
```

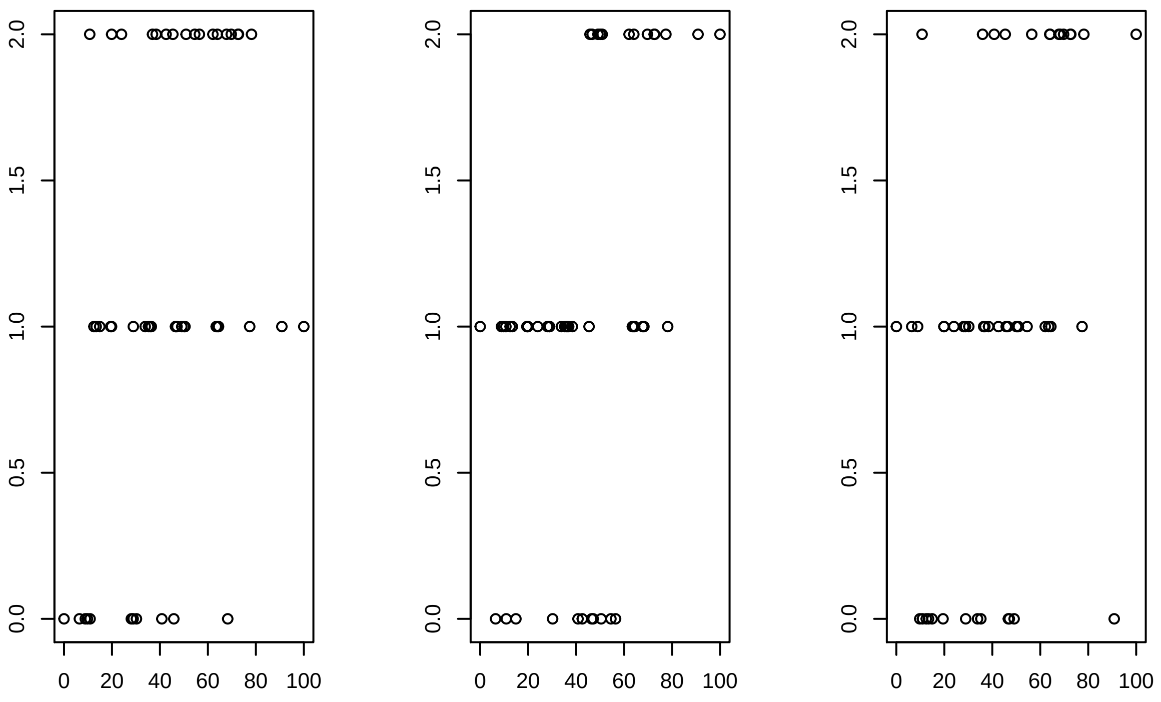


```r
par(mfrow=c(1, 1))
```

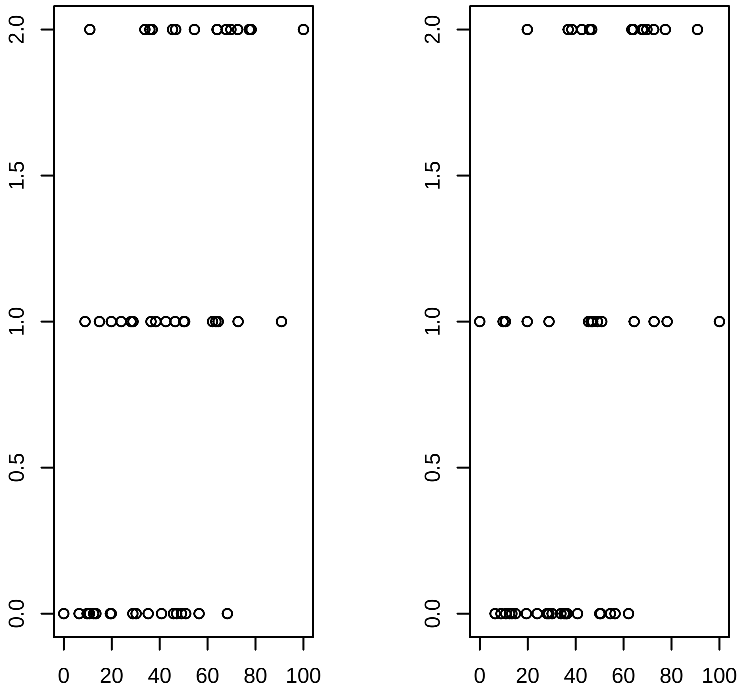

4 Create new simulated data sets

For the simulation study here, 8 populations are simulated. In the baseline climate, populations have a centred value on the environmental gradient that ranges between 5 to 75 with a distance of 10 between populations. In the future climate, populations have centred values that range between 30 and 100 with the same distance of 10.

```
baseline.means <- seq(from=5, to=75, by=10)
baseline.means
```

```
## [1]  5 15 25 35 45 55 65 75
```

```
future.means <- baseline.means + 25
future.means
```

```
## [1]  30  40  50  60  70  80  90 100
```

A resampling procedure randomly selects 1000 individuals for each population. These individuals are selected from a subset of the allele data set that are within a range of 20 around the centres of the baseline and future populations.

```
pop.mat <- data.frame(array(0, dim=c(1000, 10)))
names(pop.mat) <- paste0("L", rep(c(1:5), each=2), c(".A", ".B"))
```

4.1 Baseline populations

```r
baseline.actuals <- baseline.means

for (p in c(1:8)) {

  interval.L <- baseline.means[p] - 10
  interval.U <- baseline.means[p] + 10
  candidates.B <- AlleleData[EnvData >= interval.L, , drop=FALSE]
  EnvData1 <- EnvData[EnvData >= interval.L]
  candidates.B <- candidates.B[EnvData1 <= interval.U, , drop=FALSE]

  EnvData2 <- EnvData1[EnvData1 <= interval.U]
  baseline.actuals[p] <- mean(EnvData2)

  pop.data <- pop.mat

  set.seed(1)
  for (i in c(1:1000)) {
    cand <- candidates.B[sample(1:nrow(candidates.B), 1), ]
    for (l in 1:5) {
      if (cand[l] == 0) {
        pop.data[i, ((l-1)*2 + 1)] <- 0
        pop.data[i, ((l-1)*2 + 2)] <- 2
      }
      if (cand[l] == 1) {
        pop.data[i, ((l-1)*2 + 1)] <- 1
        pop.data[i, ((l-1)*2 + 2)] <- 1
      }
      if (cand[l] == 2) {
        pop.data[i, ((l-1)*2 + 1)] <- 2
        pop.data[i, ((l-1)*2 + 2)] <- 0
      }
    }
  }

  if (p == 1) {
    baseline.ind <- pop.data
  }else{
    baseline.ind <- rbind(baseline.ind, pop.data)
  }

}

# convert to genind object

baseline.genind <- genind(baseline.ind)
baseline.genind@pop <- factor(rep(paste0("P", c(1:8)), each=1000),
                              levels=c(paste0("P", c(1:8))))
poppr::poppr(baseline.genind)
```

```
##     Pop    N MLG eMLG      SE    H     G lambda   E.5  Hexp    Ia  rbarD
## 1    P1 1000   8    8 0.00000 2.03  7.22  0.861 0.936 0.379 0.543 0.1402
## 2    P2 1000  10   10 0.00000 2.19  7.61  0.869 0.836 0.411 0.494 0.1257
## 3    P3 1000   9    9 0.00000 2.19  8.92  0.888 0.995 0.445 0.233 0.0616
## 4    P4 1000  12   12 0.00000 2.48 11.86  0.916 0.994 0.480 0.582 0.1530
```

```
## 5    P5 1000  17   17 0.00000 2.83 16.92  0.941 0.997 0.492 0.314 0.0790
## 6    P6 1000  16   16 0.00000 2.77 15.83  0.937 0.994 0.488 0.362 0.0919
## 7    P7 1000   9    9 0.00000 2.12  7.66  0.869 0.908 0.367 0.671 0.1731
## 8    P8 1000   6    6 0.00000 1.75  5.53  0.819 0.948 0.280 0.570 0.1549
## 9 Total 8000  39   39 0.00125 3.52 28.49  0.965 0.834 0.496 0.446 0.1123
##              File
## 1 baseline.genind
## 2 baseline.genind
## 3 baseline.genind
## 4 baseline.genind
## 5 baseline.genind
## 6 baseline.genind
## 7 baseline.genind
## 8 baseline.genind
## 9 baseline.genind
```

```
# convert to genpop object
baseline.genpop <- adegenet::genind2genpop(baseline.genind)
```

```
##
##  Converting data from a genind to a genpop object...
##
## ...done.
```

```
# compare centre and mean of population ranges
rbind(baseline.means, baseline.actuals)
```

```
##                      [,1]     [,2]     [,3]     [,4]     [,5]     [,6]     [,7]
## baseline.means   5.000000 15.00000 25.00000 35.00000 45.00000 55.00000 65.00000
## baseline.actuals 9.709839 14.20781 25.88551 34.67633 44.27678 53.80678 65.95846
##                      [,8]
## baseline.means   75.00000
## baseline.actuals 72.38733
```

Plots of the frequency of the A allele along the environmental gradient show that generally the frequency increases along the gradient. However, the increase is not monotonous for four out of five of the loci. For example, for the second locus, the frequency of the A allele is lower for the fourth population than the third populations.

As a consequence of the overlap in occurrence of A and B alleles along the gradient, frequencies lower than 0.15 are not observed.

```
freq.plot <- adegenet::makefreq(baseline.genpop)
```

```
##
##  Finding allelic frequencies from a genpop object...
##
## ...done.
```

```
par(mfrow=c(1, 5))

for (l in 1:5) {
  plot(freq.plot[, (l-1)*2 + 1], ylim=c(0, 1),
       type="b", col="red", ann=FALSE)

}
```

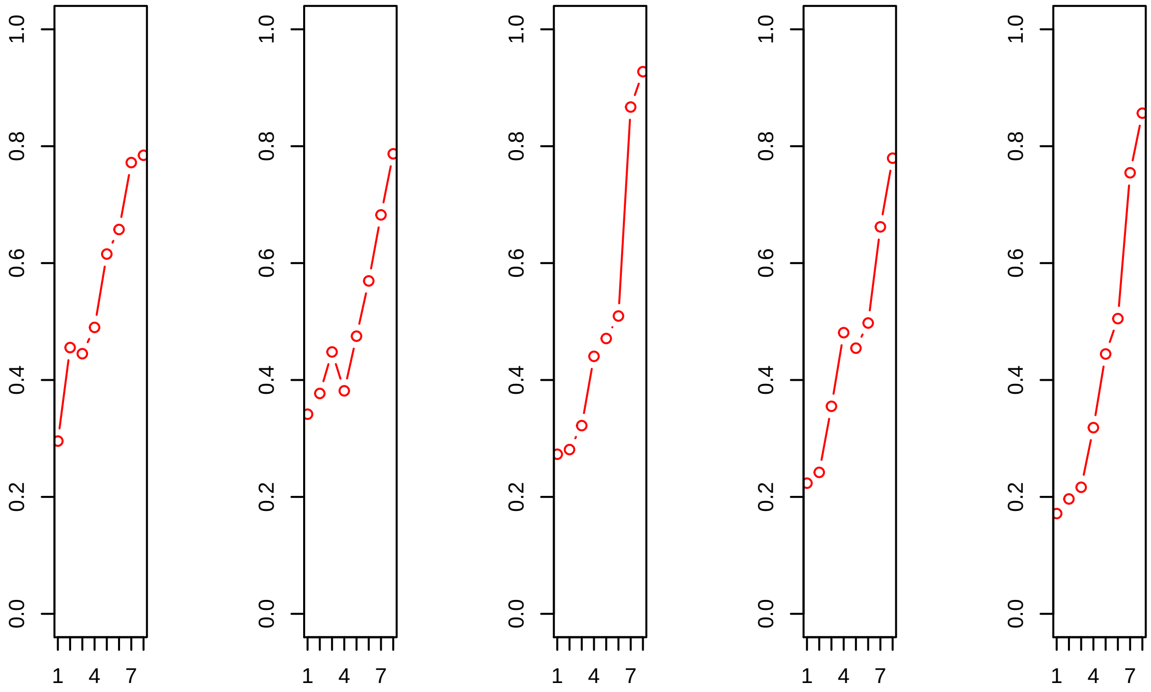


```
par(mfrow=c(1, 1))
```

4.2 Future populations

The same procedure is used to select individuals for future populations.

```
future.actuals <- future.means

for (p in c(1:8)) {

  interval.L <- future.means[p] - 10
  interval.U <- future.means[p] + 10
  candidates.B <- AlleleData[EnvData >= interval.L, , drop=FALSE]
  EnvData1 <- EnvData[EnvData >= interval.L]
  candidates.B <- candidates.B[EnvData1 <= interval.U, , drop=FALSE]

  EnvData2 <- EnvData1[EnvData1 <= interval.U]
  future.actuals[p] <- mean(EnvData2)

  pop.data <- pop.mat

  set.seed(1)
  for (i in c(1:1000)) {
    cand <- candidates.B[sample(1:nrow(candidates.B), 1), ]
    for (l in 1:5) {
      if (cand[l] == 0) {
        pop.data[i, ((l-1)*2 + 1)] <- 0
```

```r
        pop.data[i, ((l-1)*2 + 2)] <- 2
      }
      if (cand[l] == 1) {
        pop.data[i, ((l-1)*2 + 1)] <- 1
        pop.data[i, ((l-1)*2 + 2)] <- 1
      }
      if (cand[l] == 2) {
        pop.data[i, ((l-1)*2 + 1)] <- 2
        pop.data[i, ((l-1)*2 + 2)] <- 0
      }
    }
  }

  if (p == 1) {
    future.ind <- pop.data
  }else{
    future.ind <- rbind(future.ind, pop.data)
  }

}

# convert to genind object

future.genind <- genind(future.ind)
future.genind@pop <- factor(rep(paste0("P", c(1:8)), each=1000),
                            levels=c(paste0("P", c(1:8))))
poppr::poppr(future.genind)
```

```
##     Pop    N MLG eMLG      SE     H     G lambda   E.5  Hexp    Ia  rbarD
## 1    P1 1000  11   11 0.00000 2.393 10.89  0.908 0.995 0.464 0.327 0.0876
## 2    P2 1000  15   15 0.00000 2.702 14.83  0.933 0.994 0.495 0.489 0.1233
## 3    P3 1000  13   13 0.00000 2.559 12.84  0.922 0.994 0.486 0.370 0.0947
## 4    P4 1000  12   12 0.00000 2.471 11.64  0.914 0.982 0.455 0.479 0.1239
## 5    P5 1000  10   10 0.00000 2.240  8.77  0.886 0.926 0.343 0.512 0.1309
## 6    P6 1000   4    4 0.00000 1.384  3.98  0.749 0.997 0.243 0.624 0.1561
## 7    P7 1000   2    2 0.00000 0.693  2.00  0.500 1.000 0.350 1.667 1.0000
## 8    P8 1000   2    2 0.00000 0.693  2.00  0.500 1.000 0.350 1.667 1.0000
## 9 Total 8000  35   35 0.00654 3.271 18.75  0.947 0.701 0.466 0.420 0.1056
##            File
## 1 future.genind
## 2 future.genind
## 3 future.genind
## 4 future.genind
## 5 future.genind
## 6 future.genind
## 7 future.genind
## 8 future.genind
## 9 future.genind
```

```r
# convert to genpop object
future.genpop <- adegenet::genind2genpop(future.genind)
```

```
##
##  Converting data from a genind to a genpop object...
```

```
##
## ...done.
```

```r
# compare centre and mean of population ranges
rbind(future.means, future.actuals)
```

```
##                    [,1]     [,2]     [,3]     [,4]    [,5]    [,6]     [,7]
## future.means   30.00000 40.00000 50.00000 60.00000 70.0000 80.0000 90.00000
## future.actuals 32.43084 40.72407 48.17366 60.47149 68.7261 75.2329 95.42048
##                     [,8]
## future.means   100.00000
## future.actuals  95.42048
```

Graphs of future populations also show a general increase in the frequencies of the A allele. However, as a result from high frequencies of heterozygous individuals at the highest values of the environmental gradient for some loci, there is a significant drop in frequencies for the eight or seventh and eight population for loci 1 and 3.

```r
freq.plot <- adegenet::makefreq(future.genpop)
```

```
##
##  Finding allelic frequencies from a genpop object...
##
## ...done.
```

```r
par(mfrow=c(1, 5))

for (l in 1:5) {
  plot(freq.plot[, (l-1)*2 + 1], ylim=c(0, 1),
       type="b", col="red", ann=FALSE)
}
```

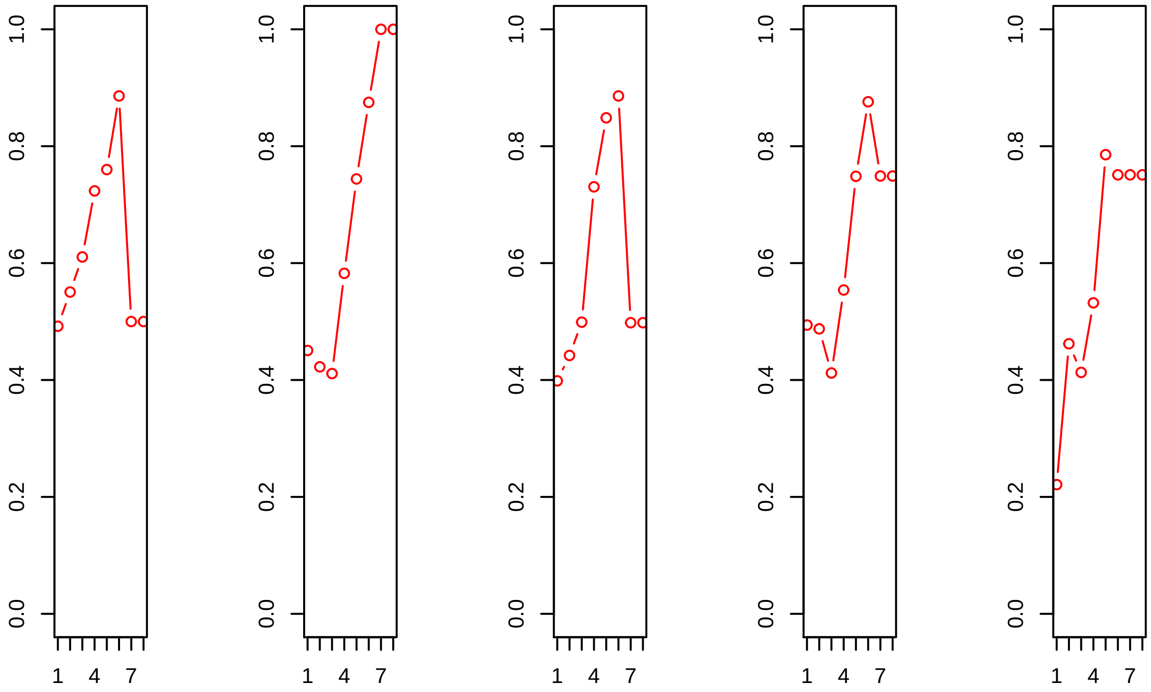


```
par(mfrow=c(1, 1))
```

Checking for novel conditions with a newer function in the package shows that novel conditions are encountered for populations 6, 7 and 8.

```
env.baseline <- data.frame(gradient=baseline.actuals)
env.future <- data.frame(gradient=future.actuals)

environmental.novel(env.baseline, env.future)
```

```
##   Pop      Var      Min     Mean      Max       SD Future.val Novel  Novel.stat
## 1   1     <NA>       NA       NA       NA       NA         NA FALSE         Inf
## 2   2     <NA>       NA       NA       NA       NA         NA FALSE         Inf
## 3   3     <NA>       NA       NA       NA       NA         NA FALSE         Inf
## 4   4     <NA>       NA       NA       NA       NA         NA FALSE         Inf
## 5   5     <NA>       NA       NA       NA       NA         NA FALSE         Inf
## 6   6 gradient 9.709839 40.11361 72.38733 23.12045   75.23290  TRUE 0.064384908
## 7   7 gradient 9.709839 40.11361 72.38733 23.12045   95.42048  TRUE 0.008375701
## 8   8 gradient 9.709839 40.11361 72.38733 23.12045   95.42048  TRUE 0.008375701
```

5 Calibrate the model

The main calibration and prediction functions from AlleleShift are used to predict baseline allele frequencies.

```
sim.count.model <- count.model(baseline.genpop,
                               env.data=env.baseline)
```

```
##
##  Finding allelic frequencies from a genpop object...
##
## ...done.
##
##
## Call:
## rda(formula = gen.comm.b ~ gradient, data = env.data)
##
## Partitioning of variance:
##               Inertia Proportion
## Total         1792015    1.00000
## Constrained   1648160    0.91972
## Unconstrained  143855    0.08028
##
## Eigenvalues, and their contribution to the variance
##
## Importance of components:
##                           RDA1       PC1       PC2       PC3       PC4
## Eigenvalue            1.648e+06 1.019e+05 2.259e+04 1.129e+04 6.135e+03
## Proportion Explained  9.197e-01 5.689e-02 1.261e-02 6.299e-03 3.424e-03
## Cumulative Proportion 9.197e-01 9.766e-01 9.892e-01 9.955e-01 9.989e-01
##                            PC5
## Eigenvalue            1.899e+03
## Proportion Explained  1.060e-03
## Cumulative Proportion 1.000e+00
##
## Accumulated constrained eigenvalues
## Importance of components:
##                          RDA1
## Eigenvalue            1648160
## Proportion Explained        1
## Cumulative Proportion       1
##
## Scaling 2 for species and site scores
## * Species are scaled proportional to eigenvalues
## * Sites are unscaled: weighted dispersion equal on all dimensions
## * General scaling constant of scores:  59.51274
##
##
## Species scores
##
##        RDA1     PC1     PC2     PC3      PC4      PC5
## L1.A -14.86  0.1611  2.5559  1.9579  1.47818  0.07634
## L1.B  14.86 -0.1611 -2.5559 -1.9579 -1.47818 -0.07634
## L2.A -13.30  3.4332  2.2391 -2.5037  0.74482 -0.09642
## L2.B  13.30 -3.4332 -2.2391  2.5037 -0.74482  0.09642
## L3.A -21.27  7.4650 -0.9318  0.8509 -0.09046 -0.80071
## L3.B  21.27 -7.4650  0.9318 -0.8509  0.09046  0.80071
## L4.A -16.61  2.3158 -2.9570 -0.2913  1.48487  0.58739
## L4.B  16.61 -2.3158  2.9570  0.2913 -1.48487 -0.58739
## L5.A -22.41  5.2760  1.0813  0.4934 -1.05359  0.93552
## L5.B  22.41 -5.2760 -1.0813 -0.4934  1.05359 -0.93552
##
```

```
##
## Site scores (weighted sums of species scores)
##
##        RDA1    PC1     PC2    PC3     PC4     PC5
## P1  25.9109  20.88  -6.643 -13.50 -39.452   2.235
## P2  21.1361  12.68  29.442  21.31  25.037   9.708
## P3  16.2412 -10.16  -1.873 -32.61  26.418 -24.735
## P4   8.0333 -11.37 -47.129  18.14  11.002   3.499
## P5   0.4916 -21.81   7.387  20.56 -10.292  19.714
## P6  -5.3667 -34.68  17.636 -14.85 -15.137   0.899
## P7 -28.8633  17.40   5.057  21.32  -8.825 -40.047
## P8 -37.5831  27.05  -3.876 -20.36  11.248  28.728
##
##
## Site constraints (linear combinations of constraining variables)
##
##      RDA1    PC1     PC2    PC3     PC4     PC5
## P1  29.58  20.88  -6.643 -13.50 -39.452   2.235
## P2  25.20  12.68  29.442  21.31  25.037   9.708
## P3  13.84 -10.16  -1.873 -32.61  26.418 -24.735
## P4   5.29 -11.37 -47.129  18.14  11.002   3.499
## P5  -4.05 -21.81   7.387  20.56 -10.292  19.714
## P6 -13.32 -34.68  17.636 -14.85 -15.137   0.899
## P7 -25.14  17.40   5.057  21.32  -8.825 -40.047
## P8 -31.40  27.05  -3.876 -20.36  11.248  28.728
##
##
## Biplot scores for constraining variables
##
##          RDA1 PC1 PC2 PC3 PC4 PC5
## gradient   -1   0   0   0   0   0
##
## Permutation test for rda under reduced model
## Permutation: free
## Number of permutations: 99
##
## Model: rda(formula = gen.comm.b ~ gradient, data = env.data)
##          Df Variance      F Pr(>F)
## Model     1  1648160 68.742   0.01 **
## Residual  6   143855
## ---
## Signif. codes:  0 '***' 0.001 '**' 0.01 '*' 0.05 '.' 0.1 ' ' 1
## Permutation test for rda under reduced model
## Terms added sequentially (first to last)
## Permutation: free
## Number of permutations: 99
##
## Model: rda(formula = gen.comm.b ~ gradient, data = env.data)
##          Df Variance      F Pr(>F)
## gradient  1  1648160 68.742   0.01 **
## Residual  6   143855
## ---
## Signif. codes:  0 '***' 0.001 '**' 0.01 '*' 0.05 '.' 0.1 ' ' 1
## Permutation test for rda under NA model
```

```
## Marginal effects of terms
## Permutation: free
## Number of permutations: 99
##
## Model: rda(formula = gen.comm.b ~ gradient, data = env.data)
##          Df Variance      F Pr(>F)
## gradient  1  1648160 68.742   0.01 **
## Residual  6   143855
## ---
## Signif. codes:  0 '***' 0.001 '**' 0.01 '*' 0.05 '.' 0.1 ' ' 1
```

```
sim.pred.baseline <- count.pred(sim.count.model,
                                env.data=env.baseline)

sim.freq.model <- freq.model(sim.pred.baseline)
```

```
##
## Family: binomial
## Link function: logit
##
## Formula:
## cbind(A, B) ~ s(Freq.e1)
##
## Parametric coefficients:
##             Estimate Std. Error z value Pr(>|z|)
## (Intercept) 0.009538   0.007938   1.202    0.229
##
## Approximate significance of smooth terms:
##              edf Ref.df Chi.sq p-value
## s(Freq.e1) 8.664  8.967  10769  <2e-16 ***
## ---
## Signif. codes:  0 '***' 0.001 '**' 0.01 '*' 0.05 '.' 0.1 ' ' 1
##
## R-sq.(adj) =  0.951   Deviance explained = 95.7%
## UBRE = 14.608  Scale est. = 1         n = 40
```

```
sim.freq.baseline <- freq.pred(sim.freq.model,
                               count.predicted=sim.pred.baseline)
```

Comparisons of input frequencies with predicted frequencies show that in most cases differences between predicted and input frequencies are smaller than 0.05. Predictions are generally worse for the fourth population and the first allele, although absolute differences are typically not larger than 0.08.

```
plot.freqs <- sim.freq.baseline[, c("Pop.index", "Allele",
                                    "Allele.freq", "Freq.e1", "Freq.e2")]

plot.freqs$freq.diff <- abs(plot.freqs$Allele.freq - plot.freqs$Freq.e2)
plot.freqs[order(plot.freqs$Allele.freq), ]
```

```
##    Pop.index Allele Allele.freq   Freq.e1   Freq.e2    freq.diff
## 14         1   L5.A      0.1715 0.1014997 0.1648966 6.603438e-03
## 24         2   L5.A      0.1965 0.1505331 0.2165325 2.003246e-02
## 34         3   L5.A      0.2165 0.2778341 0.2601839 4.368394e-02
## 13         1   L4.A      0.2235 0.2161639 0.2471556 2.365559e-02
## 23         2   L4.A      0.2420 0.2525147 0.2497567 7.756732e-03
## 12         1   L3.A      0.2730 0.1968069 0.2442019 2.879813e-02
```

```
## 22         2   L3.A      0.2810 0.2433537 0.2483680 3.263198e-02
## 1          1   L1.A      0.2955 0.3445967 0.3432206 4.772061e-02
## 44         4   L5.A      0.3185 0.3736646 0.3881340 6.963396e-02
## 32         3   L3.A      0.3220 0.3641994 0.3744802 5.248017e-02
## 11         1   L2.A      0.3415 0.3109854 0.2924763 4.902372e-02
## 33         3   L4.A      0.3550 0.3468893 0.3469618 8.038175e-03
## 21         2   L2.A      0.3770 0.3400950 0.3358926 4.110744e-02
## 41         4   L2.A      0.3815 0.4725615 0.4425719 6.107185e-02
## 42         4   L3.A      0.4405 0.4551703 0.4395946 9.053680e-04
## 54         5   L5.A      0.4445 0.4783211 0.4439190 5.809526e-04
## 3          3   L1.A      0.4450 0.4615583 0.4405745 4.425454e-03
## 31         3   L2.A      0.4480 0.4156698 0.4283111 1.968888e-02
## 53         5   L4.A      0.4545 0.4955201 0.4497379 4.762112e-03
## 2          2   L1.A      0.4555 0.3771202 0.3927369 6.276314e-02
## 52         5   L3.A      0.4710 0.5545197 0.4939215 2.292149e-02
## 51         5   L2.A      0.4750 0.5346929 0.4749789 2.110606e-05
## 43         4   L4.A      0.4810 0.4179331 0.4295019 5.149808e-02
## 4          4   L1.A      0.4900 0.5251221 0.4672411 2.275889e-02
## 63         6   L4.A      0.4975 0.5725377 0.5139233 1.642330e-02
## 64         6   L5.A      0.5050 0.5822096 0.5255244 2.052444e-02
## 62         6   L3.A      0.5095 0.6531400 0.6278525 1.183525e-01
## 61         6   L2.A      0.5695 0.5963683 0.5434386 2.606145e-02
## 5          5   L1.A      0.6155 0.5945402 0.5410653 7.443475e-02
## 6          6   L1.A      0.6575 0.6634488 0.6457116 1.178843e-02
## 73         7   L4.A      0.6620 0.6707428 0.6587942 3.205812e-03
## 71         7   L2.A      0.6825 0.6750106 0.6665957 1.590432e-02
## 74         7   L5.A      0.7545 0.7146776 0.7404661 1.403391e-02
## 7          7   L1.A      0.7720 0.7513141 0.8019730 2.997297e-02
## 83         8   L4.A      0.7795 0.7226984 0.7548489 2.465109e-02
## 8          8   L1.A      0.7845 0.7977994 0.8606631 7.616308e-02
## 81         8   L2.A      0.7870 0.7166164 0.7439786 4.302136e-02
## 84         8   L5.A      0.8565 0.7847601 0.8464747 1.002526e-02
## 72         7   L3.A      0.8670 0.7788906 0.8395316 2.746837e-02
## 82         8   L3.A      0.9275 0.8454192 0.9013085 2.619151e-02
```

```r
boxplot(freq.diff ~ Pop.index, data=plot.freqs)
abline(h=0.08, col="red")
abline(h=0.05, col="red")
abline(h=0.01, col="red")
```

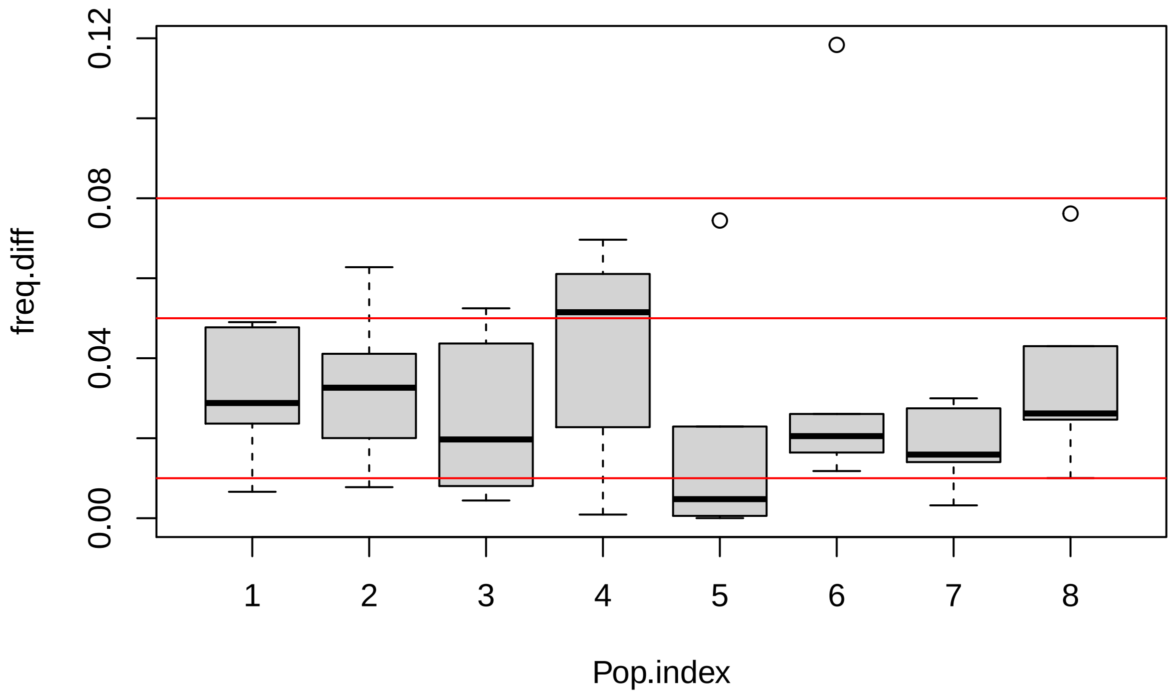


```r
boxplot(freq.diff ~ Allele, data=plot.freqs)
abline(h=0.08, col="red")
abline(h=0.05, col="red")
abline(h=0.01, col="red")
```

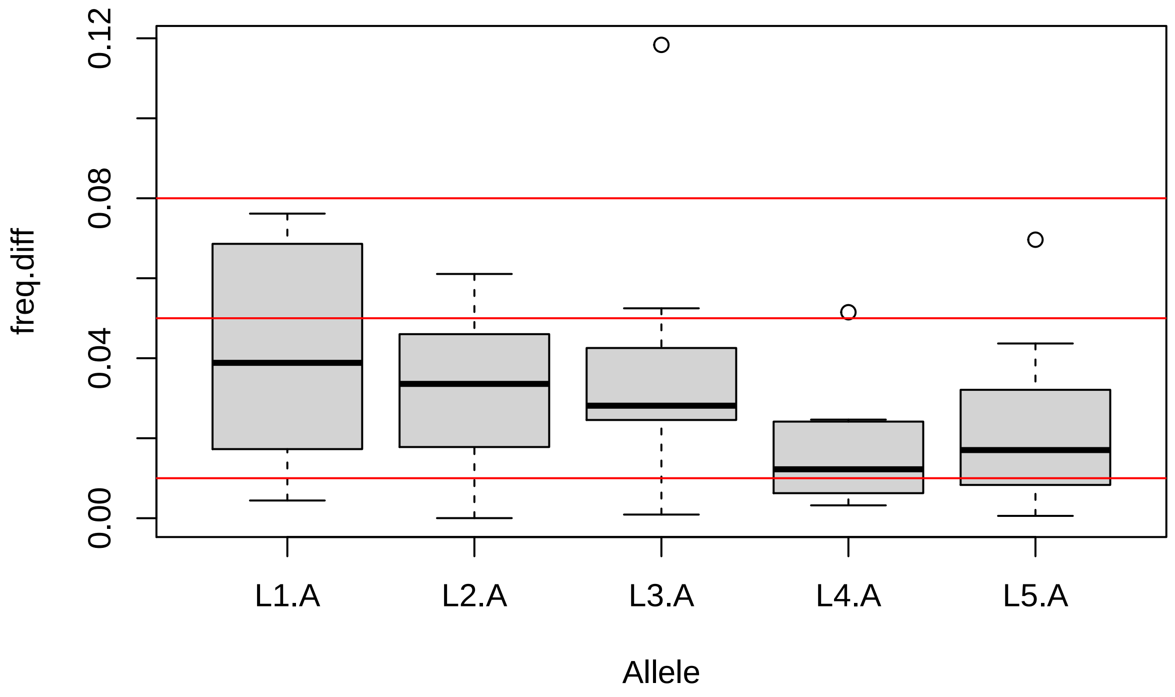

Where there is more variation in predictions between the RDA (step 1) and GAM model (step 2) between populations, there is less variation among these predictions between alleles.

```
plot.freqs$freq.diff.e <- abs(plot.freqs$Freq.e1 - plot.freqs$Freq.e2)

boxplot(freq.diff.e ~ Pop.index, data=plot.freqs)
abline(h=0.08, col="red")
abline(h=0.05, col="red")
abline(h=0.01, col="red")
```

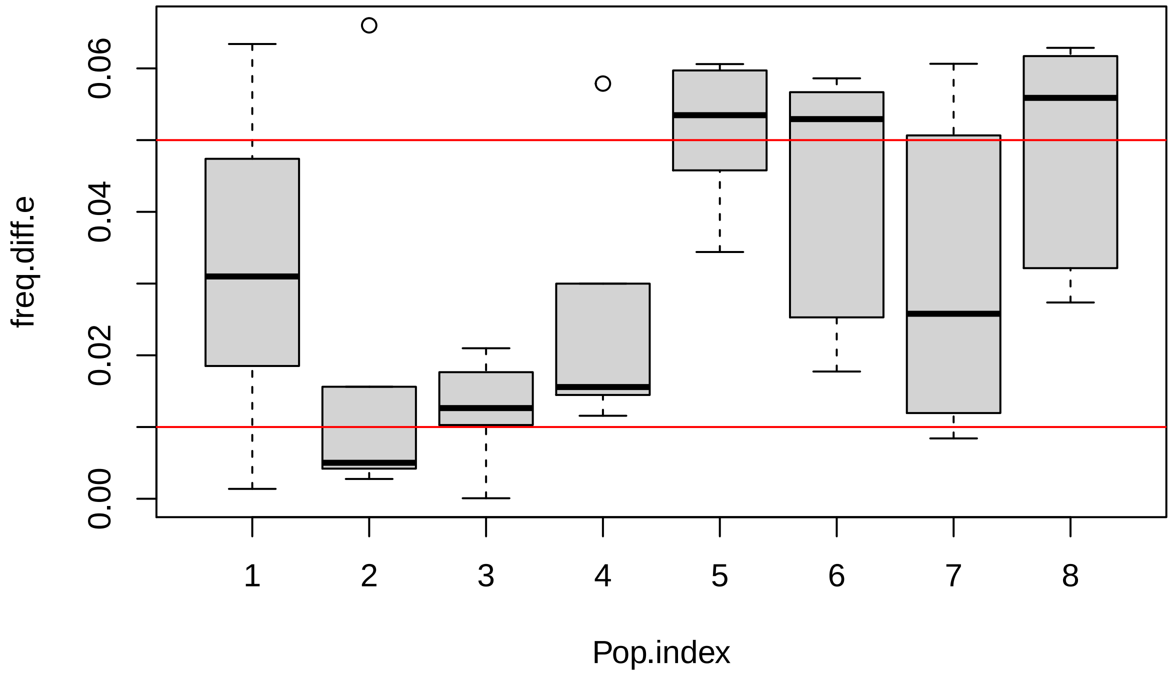


```
boxplot(freq.diff.e ~ Allele, data=plot.freqs)
abline(h=0.08, col="red")
abline(h=0.05, col="red")
abline(h=0.01, col="red")
```

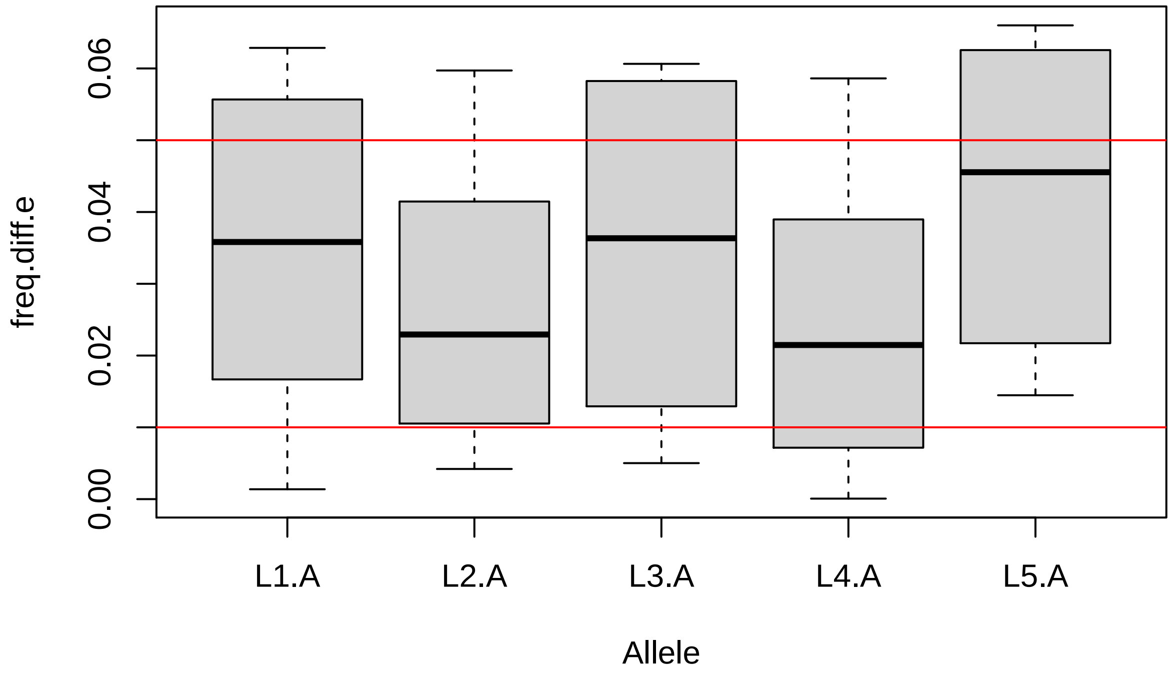

Plots of the best predictions, using a threshold of 0.50, confirm the good agreement between input and predicted frequencies.

```
plotA1 <- freq.ggplot(sim.freq.baseline,
                      colour.Pop=TRUE,
                      plot.best=TRUE,
                      threshold=0.50,
                      xlim=c(-0.05, 1.0),
                      ylim=c(0.0, 1.0))
```

```
## Populations ordered by R2
```

```
##   Pop Pop.label    N GAM.rsq
## 2  P2          2 1000    0.98
## 3  P3          3 1000    0.93
## 7  P7          7 1000    0.93
## 5  P5          5 1000    0.86
## 1  P1          1 1000    0.68
## 4  P4          4 1000    0.61
## 8  P8          8 1000    0.57
## 6  P6          6 1000    0.38
```

```
## selected populations
```

```
## [1] "P1" "P2" "P3" "P4" "P5" "P7" "P8"
```

```
plotA1
```

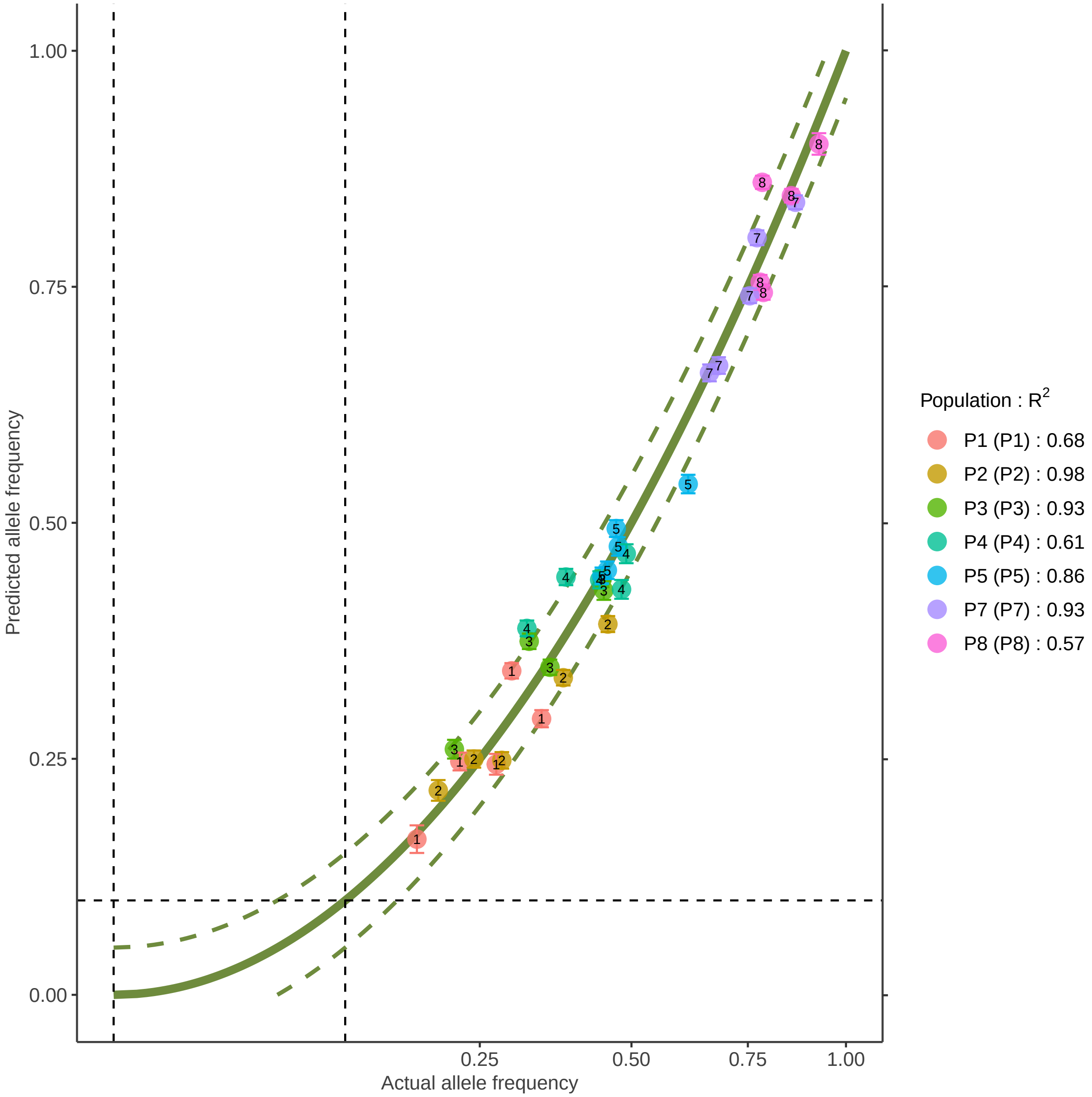

There is only one population where variation explained is less than 50 percent. This low percentage seems to be mainly a result from a narrow range in input frequencies as predicted frequencies match the overall 1:1 trend well.

```
plotA2 <- freq.ggplot(sim.freq.baseline,
                      colour.Pop=TRUE,
                      plot.best=FALSE,
                      threshold=0.50,
                      xlim=c(-0.05, 1.0),
                      ylim=c(0.0, 1.0))
```

```
## Populations ordered by R2
```

```
##   Pop Pop.label    N GAM.rsq
## 2  P2         2 1000    0.98
```

```
## 3  P3          3 1000     0.93
## 7  P7          7 1000     0.93
## 5  P5          5 1000     0.86
## 1  P1          1 1000     0.68
## 4  P4          4 1000     0.61
## 8  P8          8 1000     0.57
## 6  P6          6 1000     0.38
```

```
## selected populations
```

```
## [1] "P6"
```

```
plotA2
```

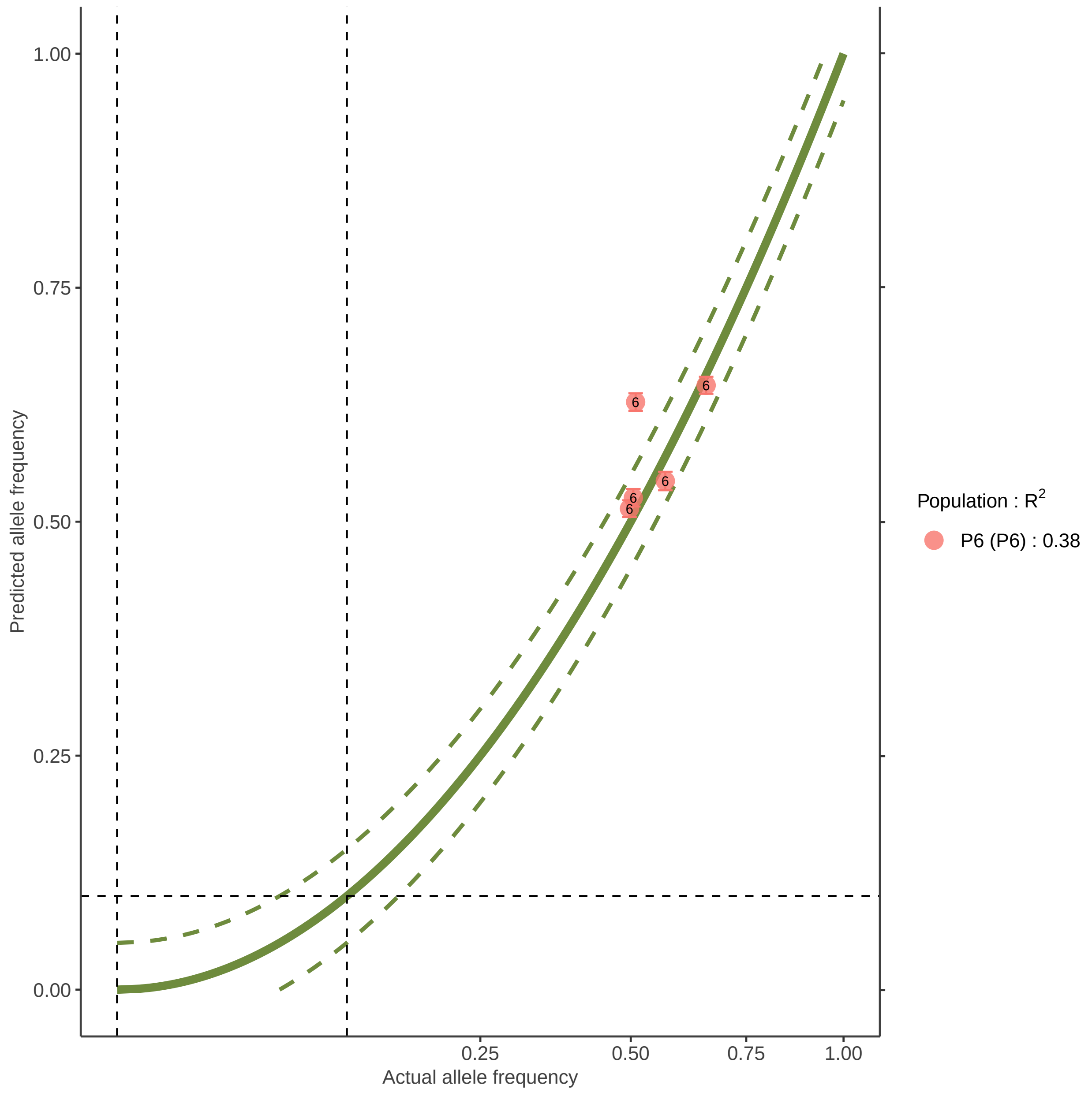

6 Predict future frequencies

Predictions for future climates are predicted with the models calibrated with the baseline data.

```
sim.pred.future <- count.pred(sim.count.model,
                              env.data=env.future)

sim.freq.future <- freq.pred(sim.freq.model,
                             count.predicted=sim.pred.future)
```

6.1 Compare predicted and simulated future frequencies

The model calibrated in this section will only be used to estimate the actual future allele frequencies. These actual allele frequencies are then used for plotting actual versus predicted (using the model calibrated with baseline frequencies) frequencies in the following figure.

```
# new calibration, just to have the input frequencies

sim.count.model2 <- count.model(future.genpop,
                                env.data=env.future)
```

```
##
##  Finding allelic frequencies from a genpop object...
##
## ...done.
##
##
## Call:
## rda(formula = gen.comm.b ~ gradient, data = env.data)
##
## Partitioning of variance:
##               Inertia Proportion
## Total         1546374     1.0000
## Constrained    914963     0.5917
## Unconstrained  631411     0.4083
##
## Eigenvalues, and their contribution to the variance
##
## Importance of components:
##                            RDA1       PC1       PC2       PC3       PC4
## Eigenvalue            9.150e+05 5.228e+05 7.337e+04 2.972e+04 5.551e+03
## Proportion Explained  5.917e-01 3.381e-01 4.745e-02 1.922e-02 3.590e-03
## Cumulative Proportion 5.917e-01 9.297e-01 9.772e-01 9.964e-01 1.000e+00
##                             PC5
## Eigenvalue            4.829e-02
## Proportion Explained  3.123e-08
## Cumulative Proportion 1.000e+00
##
## Accumulated constrained eigenvalues
## Importance of components:
##                          RDA1
## Eigenvalue             914963
## Proportion Explained        1
## Cumulative Proportion       1
##
```

```
## Scaling 2 for species and site scores
## * Species are scaled proportional to eigenvalues
## * Sites are unscaled: weighted dispersion equal on all dimensions
## * General scaling constant of scores:  57.35921
##
##
## Species scores
##
##          RDA1     PC1     PC2     PC3     PC4        PC5
## L1.A  -0.8443 -13.188 -1.7036  1.6404 -1.7246  0.0017742
## L1.B   0.8443  13.188  1.7036 -1.6404  1.7246 -0.0017742
## L2.A -22.5031  -1.437  5.9015  0.7826  0.4149  0.0050758
## L2.B  22.5031   1.437 -5.9015 -0.7826 -0.4149 -0.0050758
## L3.A  -5.0967 -16.754 -1.5579  1.0117  1.5826 -0.0006249
## L3.B   5.0967  16.754  1.5579 -1.0117 -1.5826  0.0006249
## L4.A -12.3121  -6.870  6.1488 -0.2934 -0.4822 -0.0044681
## L4.B  12.3121   6.870 -6.1488  0.2934  0.4822  0.0044681
## L5.A -16.9904  -7.227 -0.2983 -5.2155 -0.1461  0.0014498
## L5.B  16.9904   7.227  0.2983  5.2155  0.1461 -0.0014498
##
##
## Site scores (weighted sums of species scores)
##
##       RDA1     PC1     PC2      PC3      PC4      PC5
## P1  29.406  14.870  32.362  20.1028  16.5216  -8.1527
## P2  21.894   8.109   2.086 -35.4683 -18.8144  26.8232
## P3  25.346   9.153 -33.033   6.0973 -18.8697 -32.9386
## P4   7.048 -10.833 -27.816  23.4865  19.8792  32.0444
## P5 -15.489 -27.331   0.211 -28.1481  30.5617 -19.7732
## P6 -24.179 -34.894  18.557  15.6634 -31.2401   0.5232
## P7 -22.013  20.463   3.816  -0.8668   0.9808   0.7369
## P8 -22.013  20.463   3.816  -0.8668   0.9808   0.7369
##
##
## Site constraints (linear combinations of constraining variables)
##
##       RDA1     PC1     PC2      PC3      PC4      PC5
## P1  29.442  14.870  32.362  20.1028  16.5216  -8.1527
## P2  21.846   8.109   2.086 -35.4683 -18.8144  26.8232
## P3  15.023   9.153 -33.033   6.0973 -18.8697 -32.9386
## P4   3.759 -10.833 -27.816  23.4865  19.8792  32.0444
## P5  -3.802 -27.331   0.211 -28.1481  30.5617 -19.7732
## P6  -9.762 -34.894  18.557  15.6634 -31.2401   0.5232
## P7 -28.253  20.463   3.816  -0.8668   0.9808   0.7369
## P8 -28.253  20.463   3.816  -0.8668   0.9808   0.7369
##
##
## Biplot scores for constraining variables
##
##          RDA1 PC1 PC2 PC3 PC4 PC5
## gradient   -1   0   0   0   0   0
##
## Permutation test for rda under reduced model
## Permutation: free
```

```
## Number of permutations: 99
##
## Model: rda(formula = gen.comm.b ~ gradient, data = env.data)
##          Df Variance      F Pr(>F)
## Model     1   914963 8.6945   0.01 **
## Residual  6   631411
## ---
## Signif. codes:  0 '***' 0.001 '**' 0.01 '*' 0.05 '.' 0.1 ' ' 1
## Permutation test for rda under reduced model
## Terms added sequentially (first to last)
## Permutation: free
## Number of permutations: 99
##
## Model: rda(formula = gen.comm.b ~ gradient, data = env.data)
##          Df Variance      F Pr(>F)
## gradient  1   914963 8.6945   0.01 **
## Residual  6   631411
## ---
## Signif. codes:  0 '***' 0.001 '**' 0.01 '*' 0.05 '.' 0.1 ' ' 1
## Permutation test for rda under NA model
## Marginal effects of terms
## Permutation: free
## Number of permutations: 99
##
## Model: rda(formula = gen.comm.b ~ gradient, data = env.data)
##          Df Variance      F Pr(>F)
## gradient  1   914963 8.6945   0.01 **
## Residual  6   631411
## ---
## Signif. codes:  0 '***' 0.001 '**' 0.01 '*' 0.05 '.' 0.1 ' ' 1
```

```
sim.pred.future2 <- count.pred(sim.count.model2,
                               env.data=env.future)

sim.freq.plot <- sim.freq.future
sim.freq.plot$Allele.freq <- sim.pred.future2$Allele.freq
```

The comparison between projected frequencies and those sampled from the environmental gradient generally shows that projections match the expected frequencies well.

As a consequence from peculiarities of the simulated data from LEA, matches are worse for populations 7 and 8, where simulated data had lower frequencies for several alleles. Predictions for these populations were close to 1.0, which conforms more to expected frequencies of alleles that are positively correlated with the environmental gradient.

```
plotB1 <- freq.ggplot(sim.freq.plot,
                      colour.Pop=TRUE,
                      plot.best=TRUE,
                      threshold=0.50,
                      xlim=c(-0.05, 1.0),
                      ylim=c(0.0, 1.0))
```

```
## Populations ordered by R2
```

```
##   Pop Pop.label    N GAM.rsq
## 3  P3         3 1000    0.89
```

```
## 4  P4         4 1000    0.88
## 1  P1         1 1000    0.78
## 5  P5         5 1000    0.65
## 7  P7         7 1000    0.60
## 8  P8         8 1000    0.60
## 2  P2         2 1000    0.46
## 6  P6         6 1000    0.03
```

```
## selected populations
```

```
## [1] "P1" "P3" "P4" "P5" "P7" "P8"
```

```
plotB1
```

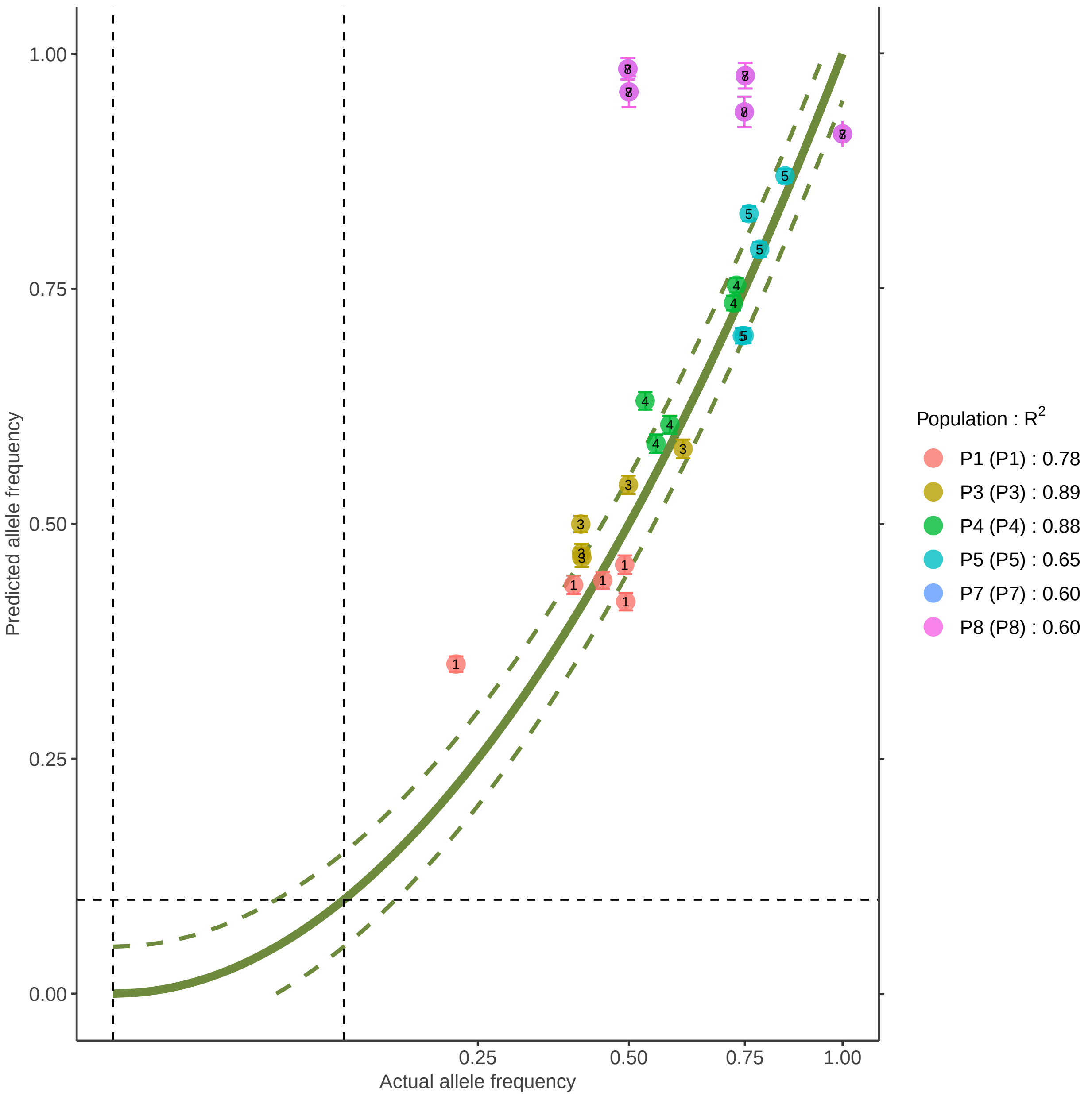

Predicted frequencies for populations 2 and 6 match the expected frequencies well. The lower percentages of explained variation are a result from the expected frequencies being close to each other, especially for

population 6.

```
plotB2 <- freq.ggplot(sim.freq.plot,
                      colour.Pop=TRUE,
                      plot.best=FALSE,
                      threshold=0.50,
                      xlim=c(-0.05, 1.0),
                      ylim=c(0.0, 1.0))
```

```
## Populations ordered by R2
```

```
##   Pop Pop.label    N GAM.rsq
## 3  P3          3 1000    0.89
## 4  P4          4 1000    0.88
## 1  P1          1 1000    0.78
## 5  P5          5 1000    0.65
## 7  P7          7 1000    0.60
## 8  P8          8 1000    0.60
## 2  P2          2 1000    0.46
## 6  P6          6 1000    0.03
```

```
## selected populations
```

```
## [1] "P2" "P6"
```

```
plotB2
```

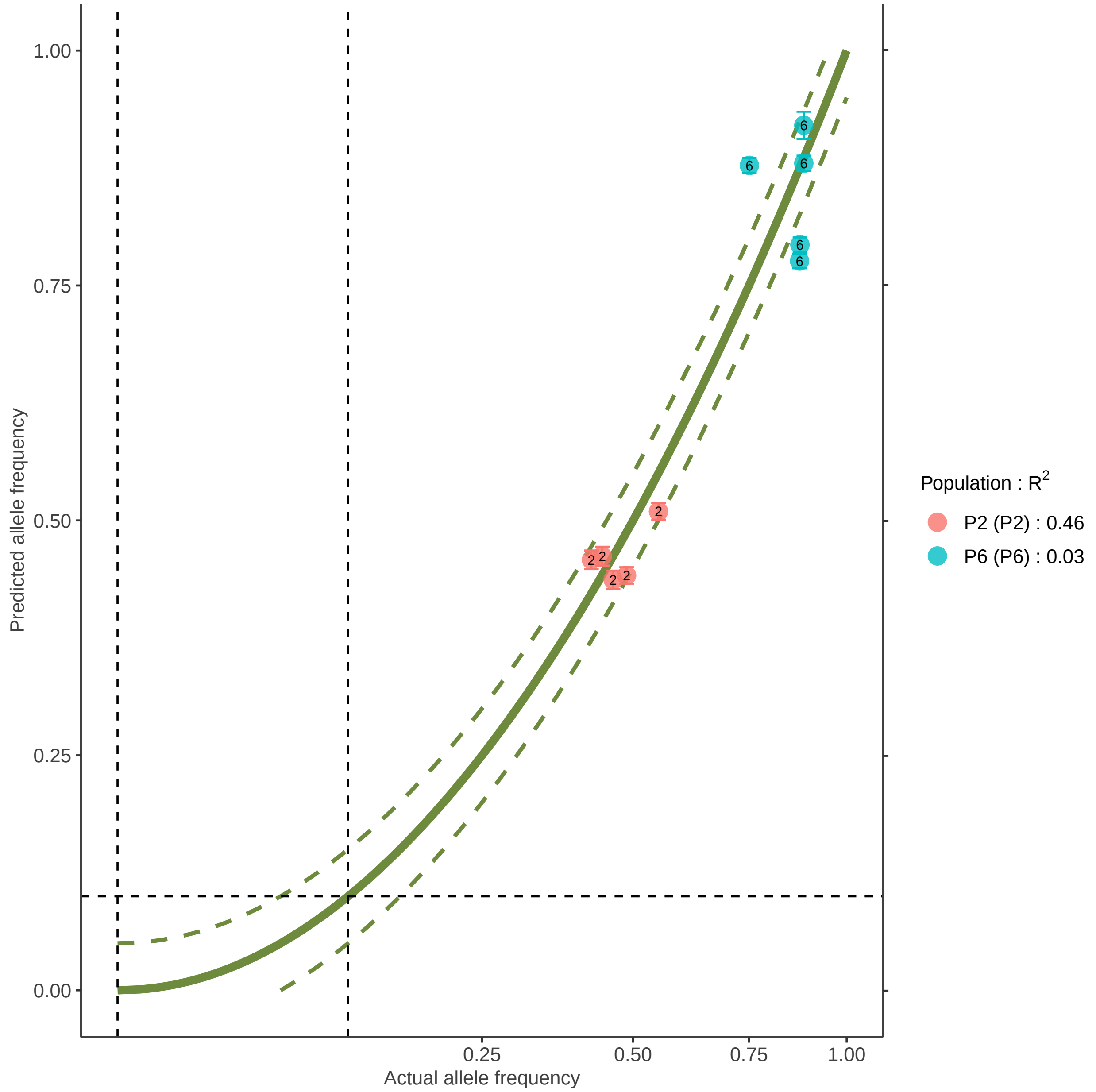

6.2 Plot predicted shifts in allele frequencies

Graphs show that increases in frequencies are expected for all populations and all loci. This is the expected trend for the simulated data.

```
ggdot1 <- shift.dot.ggplot(sim.freq.future)
ggdot1
```

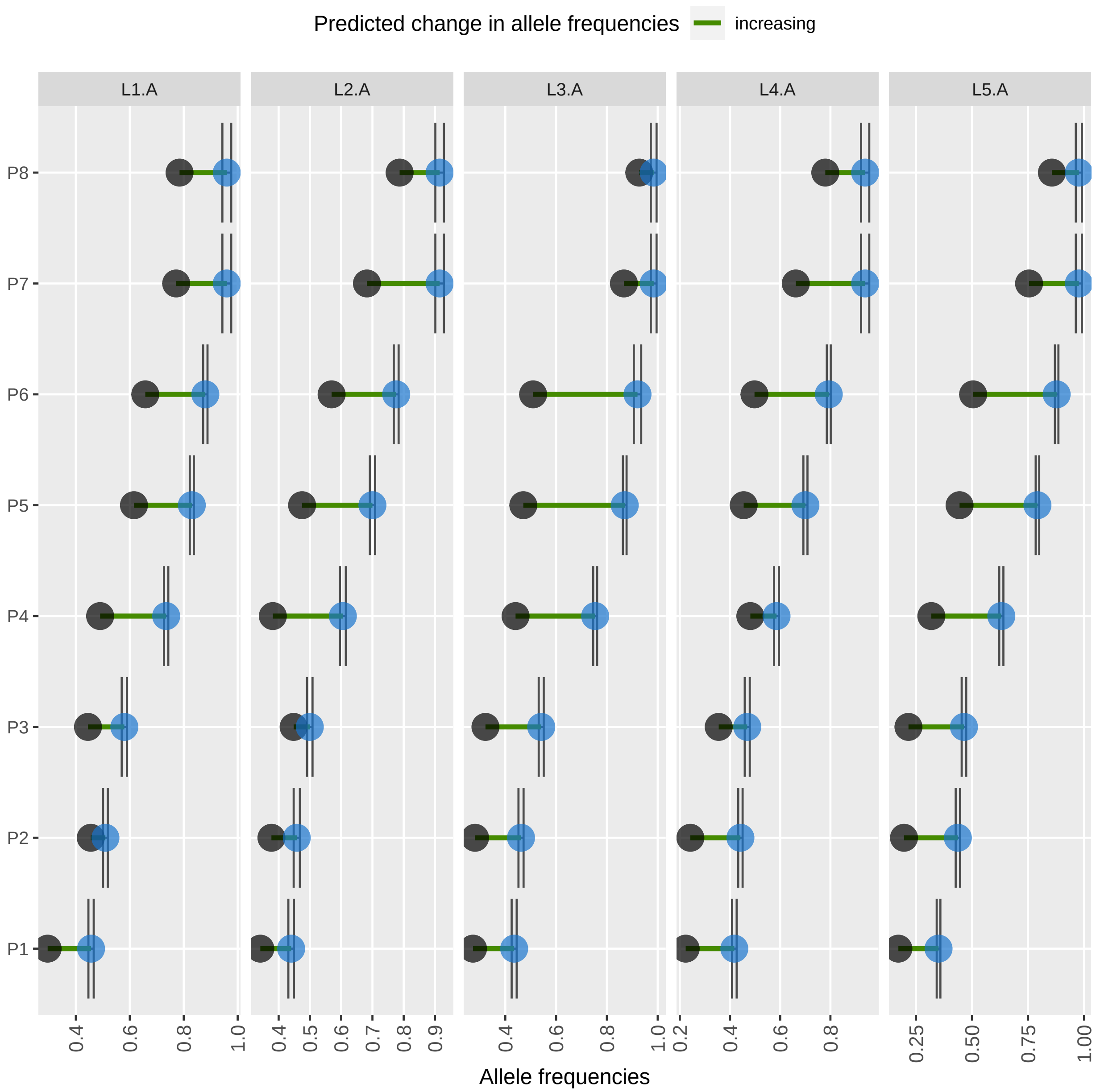


```
sim.baseline.pie <- pie.baker(sim.freq.baseline, r0=0.1)

sim.future.pie <- pie.baker(sim.freq.future, r0=0.1,
                            freq.focus="Freq.e2",
                            ypos=1)

ggpie1 <- shift.pie.ggplot(sim.baseline.pie,
                           sim.future.pie)
ggpie1
```

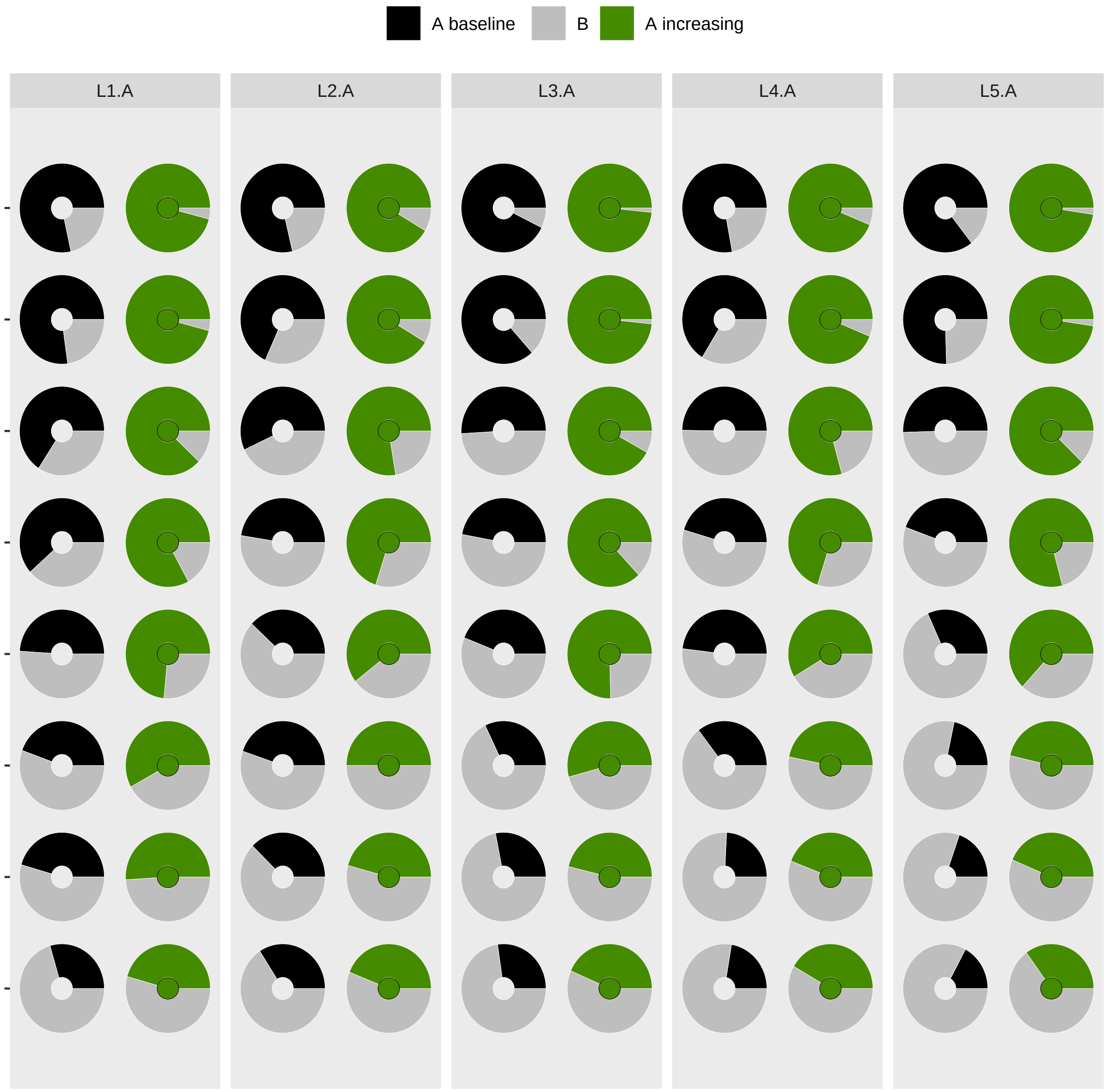

7 Discussion

The simulation procedure was able to retrieve the expected future trend of increasing allele frequencies associated with a shift of populations along the environmental gradient.

This positive trend was predicted for all populations, despite some peculiarities of the simulated data in not having monotonous increments in allele frequencies in the simulated data sets.
